# Gender Comparison of Psychological Reaction Between Breast Cancer Survivors and Their Spouses

**DOI:** 10.3389/fpsyg.2021.722877

**Published:** 2021-09-06

**Authors:** Tan Simin, Yan Jin, Zhang Aidi, Tan Xiaofang, Ruan Chunhong, Li Lezhi

**Affiliations:** ^1^Xiang Ya Nursing School, Central South University, Changsha, China; ^2^Nursing Department, Central South University Third Xiangya Hospital, Changsha, China

**Keywords:** breast neoplasms, gender difference, psycho-oncology, physiological stress, spouses

## Abstract

**Background:** Scant evidence exists among the different psychological issues between patients with breast cancer and their spouses. The objective of our study was to develop the measuring instrument testing psychological reaction and compare the difference in psychological reaction between patients with breast cancer and their spouses during the period of diagnosis and treatment.

**Method:** The semi-structured interview guideline was guided by the psychological stress model. In-depth interviews were conducted among patients with breast cancer and their spouses. Qualitative data was used to establish the item pool for the psychological reaction. Delphi method was used for item modifications. The items were conducted to find common factors through exploration factor analysis. Comparing the differences of common factors through *t*-test between patients with breast cancer and their spouses.

**Results:** Five couples were interviewed directed by the semi-structured interview guideline. About 38 items were reserved to formulate the questionnaire through the Delphi method. A total of 391 respondents (216 patients and 175 spouses) were recruited to complete the questionnaire. Two common structures were found through exploration factor analysis, which was named as reaction to role and body image change and negative coping reaction. The *t*-test found that the dimension of reaction to role and body image change (95% CI = 2.34–5.01, *p* < 0.001) reflects the difference between patients with breast cancer and their spouses.

**Conclusion:** The reactions to role and body image change between patients with breast cancer and their spouses are different during the period of diagnosis and treatment. Clinical workers should pay attention to the different reactions and help couples deal with breast cancer smoothly.

## Introduction

The cases of breast cancer in China account for 12.2% of global newly diagnosed cases, the proportion of death is 9.6%. Over 1.6 million persons are becoming patients with breast cancer every year (Fan et al., 2014). A person diagnosed with cancer is a catastrophic event to self and family members, especially for couples with breast cancer since the disease occurs in sexual organs. The prevalence of the mental disorder is highest in patients with breast cancer compared with other cancer populations (Mehnert et al., 2014). Over 30% of patients with breast cancer experience psychological distress, of which the most frequently reported problems were depression and anxiety (Heo et al., 2017). The current life stress and psychological factors resource may decrease the quality of life in patients with cancer and accelerate cancer progress.

For partners, their distress may be higher than that of patients (Hasson-Ohayon et al., 2010). The levels of state anxiety among spouses were highest when presenting for examinations and operations and decision-making (Hoellen et al., 2019). Most of the current researches focus on the psychological problems and needs of patients with breast cancer, seldom paid attention to their male partners. However, fewer social support and strategies through minimizing processing to cope with psychological distress among male partners (Lopez et al., 2012), their psychological reaction, and comparison with that of their spouses deserve further investigation.

In psychosocial oncology, men and women respond differently after being diagnosed with cancer. Women are more likely to express their emotions than men. They are more inclined to seek support and help from family members or friends, whereas men are more dependent on healthy spouses (Salander and Hamberg, 2005). The incidence of anxiety and depression in female patients with cancer is generally higher than that of male patients with cancer (Linden et al., 2012). Significant gender differences exist after cancer diagnosis of the value system. The weight of “independent” and “intellectual” to men reduced significantly, whereas the communal value paid more attention to Greszta et al. (2021).

The psychological reaction is one aspect of the psychological stress model. Cognitive reaction, emotional reaction, and psychokinesis are components of psychological reaction (Rom and Reznick, 2016). Stress response systems are stimulated by the experience of breast cancer impact of the tumor microenvironment. Individuals differ in their psychological reactions to their vulnerability to stress resources. The degree of psychological reaction in patients with breast cancer is related to mental health and result in difficulty in psychological adaptation and uncertainty in their future (Wagner et al., 2006).

The psychological reaction is not just caused by the experience of disease and treatment, it can also be affected by perceptions of one to another between couples (Manne et al., 2014). By special reasons of chemotherapy, hormonal use, and changes of body image-related mastectomy, couples with breast cancer have a high risk of developing sexual dysfunction (Farthmann et al., 2016; Gambardella et al., 2018). Marital adjustment of couples with breast cancer is faced with severe challenges (Brandao et al., 2017). Marital quality also has been proved to be related to psychological well-being (Duggleby et al., 2015) and psychological adjustment (Brandao et al., 2017) among patients with breast cancer and their partners (Gambardella et al., 2018). Studies suggested that the quality of life in couples with breast cancer are associated with psychological statuses, such as poor illness perceptions (Duggleby et al., 2015; Fanakidou et al., 2018), hope (Duggleby et al., 2015), anxiety, depression, and defense mechanisms (Hyphantis et al., 2013).

Systematic quantitative researches on the differences in psychological reactions between patients with breast cancer and their spouses are limited, and different studies reported conflicting results. A study of 150 couples consisting of women with advanced breast cancer and their husbands found that spouses reported more general psychological distress, anxiety, and depression than patients, measured by the Brief Symptom Inventory Scale (Hasson-Ohayon et al., 2010). Compared to spouses, patients with breast cancer were more likely to report anxiety, fear, difficulty in managing their emotions, and distress of self-care. When it comes to emotion among patients with breast cancer and their partners, women express more emotion than their partners when facing breast cancer in the immediate post-surgery period (Favez et al., 2017). It could be explained through gender difference which men are unwilling to express feelings or weaken emotional expression compared with that of women (Matud et al., 2003).

There are no united and standardized instruments to compare the psychological reaction of patients with breast cancer and their spouses. The purposes of this study were, first, to develop a measurement tool to test the psychological reaction of patients with breast cancer and spouses, and second, to compare the difference in psychological reaction between the patients with breast cancer and their spouses.

## Materials and Methods

### Participants and Study Design

Patients were the women who were diagnosed with breast cancer after surgery and during the period of chemotherapy, and spouses were their main caregivers. Spouses were recruited if their wives meet the above standards. All of them should have normal cognitive abilities and good communication skills. Purposive sampling was used to choose interviewees who or whose wife was receiving treatment in the hospital. In order to obtain comprehensive interview data to meet the needs of the scale, we try to choose different ages, lengths of marriage, education level, occupation, and economic status as our interview subjects. The participants understood the objective of the study and signed an informed consent agreement.

Each interview lasted about 30–60 min and was audio-tape recorded before getting their consent. Interviews stopped until the information reached saturation. To have an in-depth investigation of the problem we offer from semi-structured interview guidelines and collect more useful information, a quiet, isolated consulting room was chosen to interview each participant face to face individually and nobody else participates in the interview except the researcher. We always start the talk with a simple question to relieve the nervous atmosphere and bring it into the topic. For example, “how do you feel recently?” As the interview time went by, doubtful answers were dug deeper and some sensitive questions were asked. The interviews were recorded by digital voice recorder, and participants were informed consent. The recording was transcribed by two researchers, respectively, after the interview ends. For inconsistent transcription content, our research team reached an agreement after discussion. The interview data were analyzed using the Colaizzi phenomenological approach as described by Polit and Beck (Stannard, 2012). All of the interviews were read carefully and repeatedly to acquire a feeling for researchers. Then we coded them into word for word literally. Two researchers compare their analysis and discuss any discrepancies until reaching an agreement. The significant statements extracted from the interview was formed the item pool finally.

Experts were invited to review items and evaluated each item from the aspect of importance, familiarity, and judgment basis. Experts evaluated each item and rated it from 1 (no correlation) to 4 (strong correlation). The research group deleted or modified items based on the evaluation of experts. Items were deleted as following standards: (1) the implication item illustrated was contained in other items; (2) items were not inconsistent with the core theme we researched; (3) the meaning of the item was likely to lead to ambiguity or expressed improperly; and (4) the average score experts gave <3.0. We then modified the item pool through two rounds of the Delphi method and formulated the scale.

Each item consists of a 5-point Likert-type scale ranging from 1 (strongly disagree) to 5 (strongly agree). Rates of all items were added together to create total scores of the measurement, with higher scores indicate psychological reaction more intensively. We got the agreement of recipients and sign the informed consent statement before the data were collected in a group of patients and a group of spouses, respectively. Using exploratory factor analysis (EFA), we extract items and compare the difference of each common factor between the group of patients and their spouses.

### Statistical Methods

NVivo 10 was developed by QSR international in the United States and was used to transcribe and code qualitative interview data and establish an item pool. Item analyses were conducted to test the adequacy and reliability of items, sort the total scores, and use 27% as the demarcation point to divide into high-score group and low-score group. The differences of each item in the high-score group and low-score group were analyzed by an independent sample *T*-test. Items were reserved when statistically significant differences were found. The EFA was used by the following methods. Principal components analysis was used to extract factors, which eigenvalue was >1. Maximum iterations for convergence were 25, which was the default value set by SPSS 18.0 (SPSS 18.0 was developed by IBM in the United States). Orthogonal rotation (maximum variance method) was applied the supposition that there was no correlation among each common factor. The Pearson product–moment correlation coefficient was used to calculate the test–retest reliability of the scale. Differences of each common factor between the group of patients and group of spouses were evaluated using an independent sample *t*-test.

## Results

### Qualitative Interview

The information reached saturation until five couples were interviewed. The age of patients with breast cancer was range from 29 to 57. The average age of patients with breast cancer was 42.07 ± 9.02 years old, and 44 ± 8.67 years old for spouses. About 64 items were extracted from the qualitative data by the research group. The experts were from different fields, one statistics professor, two psychology professors, two associate professors from breast surgery, and five nursing specialists. The response rate was 90%, one of them did not reply after sending the email twice.

Six items were contained in other items, two items did not conform to the core theme we measuring, eight items were likely led to inaccurate comprehension, and 10 items experts evaluated were <3.0 on average score. About 38 items were reserved through the Delphi method finally.

### The Formation of the Questionnaire

#### Demographic Characteristics of the Participants

From January 2020 to July 2020, participants were recruited from two hospitals (Central South University, Third Xiangya Hospital, Hunan Cancer Hospital) in Changsha, Hunan, China. About 420 questionnaires were distributed to respondents, 391 questionnaires were retrieved, including 216 patients with breast cancer and 175 spouses. The effective questionnaire return ratio is 93.1%. The average age of male participants was almost 1 year older than female partners. Over 70% had received secondary education or above, more than half of the participants were farmers. There were no statistical differences between the two groups in the time of getting married, education level, and occupation (Table 1).

**Table 1:** Social-demographic variables, clinical characteristics of participants.

**Characteristic**	**Group of patients**	**Group of partners**	***p***
	***N* = 216**	***N* = 175**	
**Age (years)**	46.19 ± 8.55	47.11 ± 7.89	<0.001
**Marriage (years)**	23.05 ± 9.31	23.21 ± 8.95	0.864
**Education level**			0.816
Primary	46 (21.3%)	39 (22.3%)	
Secondary	136 (63.0%)	105 (60%)	
Tertiary	34 (15.7%)	31 (17.7%)	
**Occupation**			0.159
Farmer	127 (58.8%)	111 (63.4%)	
Industrial worker	21 (9.7%)	23 (13.1)	
Public institution worker	30 (13.9%)	21 (12.0%)	
Unemployed	22 (10.2%)	7 (4.0%)	
Others	16 (7.4%)	13 (7.4%)	

#### Psychological Reaction Scale for Patients With Breast Cancer and Their Spouses

Four methods were used to screen items in item analysis. About 27% of the participants whose score was more than 142 consisted of the high-score group, and 27% of the participants whose score was <101 were classified into the low-score group. All of the items were reserved after the *t*-test between the high-score group and the low-score group. Correlations between items and scale scores (item-total correlations) were considered adequate if each value exceeded 0.4. Five items were <0.4 to total score analyzed by the Pearson correlation coefficient (two-tailed tests at the 99% significance level). The Cronbach's α of 38-item was 0.944, and all the values of the Cronbach's α when deleted one of the items were ≤0.944. Extracting one component through principal component analysis, the variance of the common factor should exceed 0.200 or the item-factor weight should be over 0.400. Six items of the variance value did not reach the upper standard. The suitability of the data for EFA was assessed using the Kaiser–Myer–Olkin measure of sampling adequacy (0.923) and the Bartlett's test of sphericity (χ^2^ < 0.01). We deleted 14 items through EFA, and the result showed there were two component matrixes.

There were 13 items reserved to form the final scale: seven items were named “reaction to role and body image change” and six items were named “negative coping reaction” (Table 2). The Cronbach's α coefficient of the whole scale is 0.877, Cronbach's α for each dimension is over 0.700. The split-half reliability of the scale is 0.809, and for each dimension is all over 0.700. The test–retest reliability of the whole scale is 0.875. The correlation coefficient of the whole scale to “reaction to role and body image change” is 0.936 and to “negative coping reaction” is 0.894. The correlation coefficient of the two dimensions is 0.679.

**Table 2:** Exploratory factor analysis of 13 common items.

**Items**	**Components**	
	**1**	**2**
37. I cannot take any more about the change of self-body image.	**0.796**	0.140
32. I think mastectomy will reduce feminine charms.	**0.743**	0.108
26. I always dare not face the wound on the chest.	**0.642**	0.355
21. I am grieved when I see the wound on chest.	**0.618**	0.253
29. Getting ill makes me fall into despair.	**0.590**	0.404
16. I cannot accept the role as a patient with breast cancer.	**0.554**	0.432
33. The operation makes me anxious.	**0.552**	0.258
5. I am getting more and more impatient with chemotherapy.	0.090	**0.715**
7. I cannot confront sudden misfortune with optimism.	0.237	**0.701**
18. I am deliberately side-stepping the things related to the disease.	0.227	**0.699**
17. The strong side effects of chemotherapy make me unendurable.	0.223	**0.555**
22. I cannot accept the fact of chemotherapy.	0.320	**0.528**
12. The topics of cancer and breast are considered taboo for me.	0.404	**0.524**

*Values exceeding 0.4 are marked in bold and classified into corresponding components or dimensions for the convenience of readers*.

### Difference Comparison

The psychological reaction scale for the patients and spouses found two common factors through EFA among 13 items: the average score of “reaction to role and body image change” in the group of patients is 23.15, which is 3.68 higher than the group of spouses (19.47). The difference between patients and spouses was statistically significant in the dimension of “reaction to role and body image change” (95% CI = 2.34–5.01, *p* < 0.001) through the *t*-test. In the dimension of “negative coping reaction,” no obvious gender difference from data analysis was found (Table 3).

**Table 3:** Gender difference in two dimensions of psychological reaction.

	**Score (mean** ± **SD)**		***t*** **-test**			
**Dimension**	**Group of patients (*n* = 216)**	**Group of spouses (*n* = 175)**	***t***	**Lower 95% CI**	**Upper 95% CI**	***p***
Reaction to role and body image change	23.15 ± 7.09	19.47 ± 6.09	5.43	2.34	5.01	<0.001
Negative coping reaction	17.84 ± 5.54	16.81 ± 5.19	1.91	−0.03	2.11	0.057

## Discussion

The psychological reaction scale has 13 items and consists of two dimensions named “reaction to role and body image change” and “negative coping reaction.” The Cronbach's α coefficient of the scale is 0.877. The split-half reliability of the scale is 0.809, and the test–retest reliability is 0.875. These data show that the scale has good reliability and validity. It can be used to evaluate the psychological reaction of patients with breast cancer during chemotherapy after mastectomy and their spouses. It is important evidence for the clinical workers who can take specific psychological interventions to couples with breast cancer based on the results of the scale. They can focus on the different effects of changes in role and body image on patients and their spouses, helping them understand each other in depth and better cope with the adverse effects of diseases and treatments together.

In our study, it showed differences in “reaction to role and body image change” between patients and their spouses (*t* = 5.34, *p* < 0.001). Han et al. (2010) defined that characteristics of the mental image of the body of an individual, attitude about body appearance and state of health, and sexual functioning consist of body image among patients with breast cancer (Stannard, 2012). Body image is an important part of sexuality. Body alternation in body image during the treatment affects sexual response, sexual role, and relationship (Pelusi, 2006). Besides absent breast(s) and wounds or scars on the chest, 85% of patients with breast cancer experience alopecia, and 67% of the loss of eyebrows or eyelashes during the treatment (Pierrisnard et al., 2018). Age and treatment type are the important factors that impact body image, and sexual problems among patients with breast cancer (50 years or younger) are related to the poor body image (Paterson et al., 2016). Patients with breast cancer who received modified radical mastectomy had a higher frequency of altered body image (Monteiro-Grillo et al., 2005). Although the alterations in body image had a psychological effect on spouses of patients with breast cancer to some extent, patients with breast cancer were caring more about their altered body image than their partners (Holmberg et al., 2001) and often took for granted that their husbands would be repulsed by alteration of body image (Sheppard and Ely, 2008). On the contrary, spouses of patients with breast cancer stated that altered body image was secondary to the health and life of their wives (Holmberg et al., 2001). Most husbands thought lost breast or scar caused by mastectomy did not influence their relationship (Hoga et al., 2008), whereas patients with breast cancer thought exactly the opposite (Kocan and Gursoy, 2016). Identifying and clarifying perceptions of body image might reduce the difference between couples with breast cancer.

To the problems of role adaption, the reactions to role change between patients with breast cancer and their spouses are different. Patients with breast cancer are more intense than their spouses when they are faced with cancer diagnosis and treatment. It is perhaps that they are the direct victims of breast cancer, and their psychological states are more serious than their caregivers. Research has verified that patients with breast cancer experience more role problems than their spouses when compared with couples of benign breast diseases (Northouse et al., 1998). But it is undeniable that breast cancer disease has an enormous influence on role change among husbands of patients with breast cancer. Social recognition of the role of caregiver is an important experience to male spouses in the context of breast cancer from either positive or negative aspects. It means complex negotiation and the great acceptance of the role as caregiver. The balance between social roles and domestic roles was broken, role strains in the social domain improved, and in the domestic domain worsened. These role problems of male caregivers are more likely to cause psychological distress and may result in a decrease in social support and quality of life (Wagner et al., 2011).

Concerning negative emotions between patients with breast cancer and their spouses, no significant differences were found in our study. However, the current researches have controversial conclusions. Spouses reported more psychological distress than patients with breast cancer which included depression and anxiety in the study (Hasson-Ohayon et al., 2010), whereas the research results were opposite reported by Ben-Zur et al. (2001). Expressed emotion was more likely to appear in women with breast cancer when compared with their partners and highly related to the anxiety of patients with breast cancer (Matud et al., 2003). Patients who were diagnosed with breast cancer report difficulty in manage their emotions and are more likely to feel anxious and fearful. But there is no difference between patients and partners in feeling depression (Kauffmann et al., 2016). In contrast with some other findings reported (Alacacioglu et al., 2009), depression of patients with breast cancer was higher than their partners, whereas anxiety measured through the State-Trait Anxiety Inventory scale has no obvious difference between couples with breast cancer. These perhaps attribute to sample selection, different cultural backgrounds, or research methods. Items in our study probably cannot summarize negative emotion comprehensively, and it is hard to provide powerful evidence. Further researches need to verify the conclusions.

## Limitations

Our study has several limitations. The sample size of the group of spouses is under our expectations. We found it difficult to recruit male spouses to accomplish our psychometric questionnaire when compared with their wives with breast cancer. Perhaps influenced by Chinese traditional culture, some husbands may be unwilling to self-disclose their psychological problems in public. They may choose to weaken the expression of emotion and are less likely to seek out help from others (Matud et al., 2003). Economic condition, physical and emotional status of patients with breast cancer, and ethical factors could affect the likelihood of participation of spouses (Christie et al., 2013). We did not make a further investigation to analyze the reasons spouses are unwilling to participate in research in consideration of ethics. In addition, we did not use any other instruments to evaluate some differences that may be caused by psychological or personality characteristics rather than gender. In future research, we will include these factors that may lead to differences in psychological responses between the two groups and have excluded their influence on the results of the study.

## Conclusion

This study demonstrated that both patients with breast cancer and their spouses experience psychological distress during the diagnosis and treatment of breast cancer. Patients with breast cancer are more reflected in ego-defense, emotion, reaction to the spousal relationship, body image change, and chemotherapy. Partners of patients with breast cancer manifest as escapism, body image change reaction, emotion stress reaction, negative emotion, and coping response. Although altered body image caused by breast cancer has a great influence on both patients and spouses, gender differences are existing between them. These suggest that the psychological impact of body image alteration and role adaption to patients with breast cancer are more serious than their partners, intervention should be paid attention to the special phenomenon. Future studies are warranted to take psychological interventions to patients with breast cancer and their spouses, respectively, and improve the psychological status of not just patients with breast cancer but also their partners during the period of diagnosis and treatment.

## Data Availability Statement

The raw data supporting the conclusions of this article will be made available by the authors, without undue reservation.

## Ethics Statement

The studies involving human participants were reviewed and approved by The IRB of Third Xiangya Hospital, Central South University. The patients/participants provided their written informed consent to participate in this study.

## Author Contributions

YJ conceived the study and designed research methods. TX and RC collected the qualitative research data. ZA was in charge of the quantitative data collection. TS analyzed the data and written the manuscript under the guidance of LL. All authors contributed to the article and approved the submitted version.

## Conflict of Interest

The authors declare that the research was conducted in the absence of any commercial or financial relationships that could be construed as a potential conflict of interest.

## Publisher's Note

All claims expressed in this article are solely those of the authors and do not necessarily represent those of their affiliated organizations, or those of the publisher, the editors and the reviewers. Any product that may be evaluated in this article, or claim that may be made by its manufacturer, is not guaranteed or endorsed by the publisher.
